# Domain-Based Charge-Transfer Decomposition and Its Application to Explore the Charge-Transfer Character in Prototypical Dyes

**DOI:** 10.1021/acs.jctc.5c00186

**Published:** 2025-04-29

**Authors:** Lena Szczuczko, Marta Gałyńska, Maximilian H. Kriebel, Paweł Tecmer, Katharina Boguslawski

**Affiliations:** † Institute of Physics, Faculty of Physics, Astronomy, and Informatics, Nicolaus Copernicus University in Toruń, Grudziadzka 5, 87-100 Toruń, Poland; ‡ Faculty of Chemistry, Nicolaus Copernicus University in Toruń, Gagarina 7, 87-100 Toruń, Poland

## Abstract

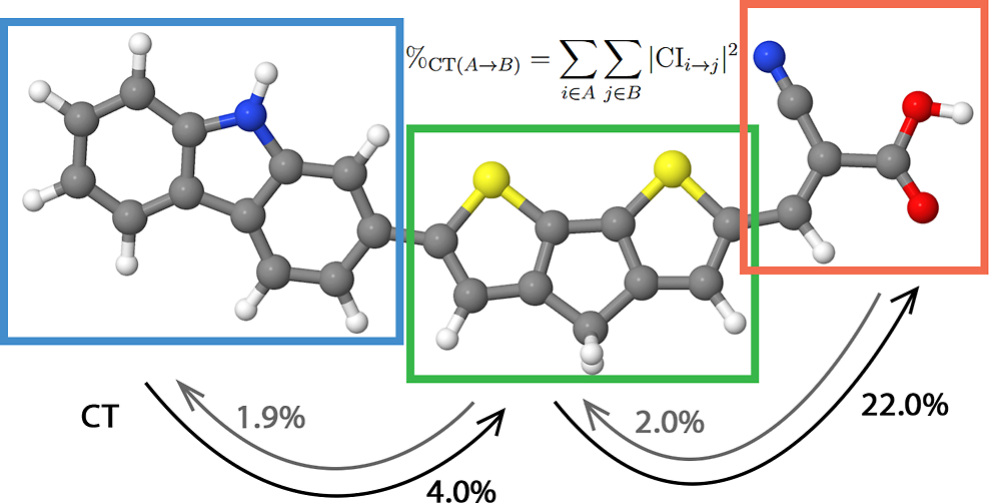

We introduce a new domain-based charge-transfer analysis tool exploiting the locality of pair Coupled Cluster Doubles orbitals. Unique features of the proposed model include the ability to monitor the direction of the charge flow between different parts or moieties of the system and its quantitative evaluation. We assess the predictive power of our new method for selected dye candidates of dye-sensitized solar cells with different doping and structural arrangements and compare our results for excitation and orbital energies to various density functional approximations and the domain-based local pair natural orbital variant of coupled cluster singles doubles. Our work confirms that the dyes with S-doped bridges are the most promising candidates for dye-sensitized solar cells applications, featuring the largest donor → bridge → acceptor directed charge transfer and the most favorable electrodonating and electroaccepting powers.

## 1 Introduction

Organic electronics has emerged as a rapidly growing field, harnessing the unique properties of organic molecules to develop novel electronic devices and technologies, from flexible displays and solar cells to sensors and bioelectronics.^1−8^ These materials offer unique advantages, including low-cost manufacturing, mechanical adaptability, and tunable optoelectronic properties. The key to their functionality is charge transfer, a critical phenomenon that governs energy and electron transport across molecules.

Developing new and even more efficient organic devices heavily relies on the fundamental understanding of the charge transfer process between the donor and acceptor units and their mutual alignment.^9^ These processes occur at the molecular level, which can be challenging to investigate experimentally. On the other hand, accurately modeling charge transfer processes presents a formidable computational challenge. Traditional methods using canonical delocalized orbitals often struggle to capture the nuanced electronic interactions that define charge transfer. This limitation is particularly evident in complex organic systems, where electron correlation effects and complex electronic structures require sophisticated computational approaches. While Density Functional Theory (DFT)^10,11^ has traditionally been the standard approach in organic electronics, it faces challenges in describing extended π-conjugated systems.^12,13^ Furthermore, DFT methods often struggle with predicting charge transfer excitation energies^14^ and suffer from delocalization errors that lead to inaccurate electron and hole densities.^13,15−23^

To that end, reliable and efficient computational methods are needed to model electronic structures and properties of the building blocks of organic electronic materials. In contrast, pair Coupled Cluster Doubles (pCCD)^24,25^ and related geminal-based methods^24−68^ have shown potential to overcome these limitations. By providing a more accurate representation of the correlated wave function, these methods offer a balanced treatment of static and dynamic correlation effects^69,70^ essential for modeling organic electronic materials.^71^

Over the past decades, several charge transfer models have been proposed to monitor and quantify the charge transfer process in molecular systems.^72−74^ Apart from the models based on the Marcus theory^75^ itself, many quantum chemical tools have been introduced to assess the degree of charge transfer in ground-state molecular complexes^76,77^ and in electronically excited states.^78^ While most of these models are DFT-based, there are also approaches dedicated specifically to wave function methods. Examples are natural transition orbitals,^79^ models based on the transition density matrix,^80^ and nonorthogonal descriptions of diabatic wave functions.^81^

In this work, we present a simple and intuitive charge transfer analysis, which breaks down electronic transitions to disjoint moieties or domains. By focusing on a moiety-based charge-transfer decomposition, we can resolve electronic transitions spatially. We will apply the proposed charge-transfer breakdown to investigate the electronic structures and excited-state properties of organic dyes. The localized nature of the natural pCCD molecular orbitals facilitates a spatial decomposition of electronic transitions, while the electronic energies and properties are in good agreement with more elaborate and expensive Coupled Cluster (CC) methods.^82^ Hence, pCCD-based approaches allow for an inexpensive, but reliable treatment of electron correlation effects crucial to modeling charge transfer processes.^71^

This work is organized as follows: In Section 2, we introduce a domain-based charge-transfer decomposition based on a pCCD reference function. Section 3 describes the computational details. Our decomposition scheme is then applied to investigate the charge-transfer character in eight dyes used in dye-sensitized solar cells (DSSC) (Section 4). We further compare the excitation energies, ionization potentials, and electron affinities obtained from various pCCD-based methods to CC Singles Doubles (CCSD) and DFT data. Finally, we conclude in Section 5.

## 2 Methodology

### 2.1 pCCD Ansatz

pCCD represents an inexpensive quantum chemistry method derived from the traditional CC approach.^24,25,33,34^ Unlike standard CCD methods that consider all possible double electron excitations, pCCD simplifies the ansatz, instead strategically focusing only on paired electron excitations. The pCCD wave function can be formulated through an exponential ansatz^25,34,37^
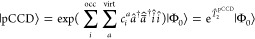
1where |Φ_0_⟩ is some reference wave function (not necessarily the Hartree–Fock determinant) and  is the cluster operator containing electron pair-excitations. In the above equation,  and  refer to the electron creation and annihilation operators, and *a*/*i* and *a̅*/*i̅* represent spin-up (α) and spin-down (β) electrons, respectively. *c*_*i*_^*a*^ denotes the pCCD cluster amplitudes, where the sum runs over all occupied *i* and virtual *a* orbitals. Furthermore, the pCCD molecular orbitals (which are used to define the reference determinant of eq 1) are typically optimized using a variational orbital optimization protocol as described in refs (34, 37 and 38). The corresponding orbital gradient of the orbital-optimized pCCD state equals zero and is determined from
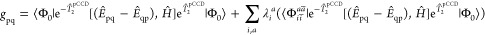
2where  is the singlet excitation operator and *p*, *q* label all active (occupied and virtual) orbitals, while λ_*i*_^*a*^ are the Lagrange multipliers determined from the pCCD Λ equations, *Ĥ* is the molecular Hamiltonian, and  encodes a pair-excited Slater determinant, and .^34,37,38^ The final natural pCCD-optimized orbitals are usually localized. The pCCD model reduces computational costs by targeting only electron-pair excitations and simultaneously enhances the accuracy of modeling strongly correlated systems, making it suitable for investigating systems with (quasi-)degeneracies, like extended organic compounds.^20,83^

While pCCD allows us to model ground-state electronic structures,^33,36−38,84^ it can be extended with the Equation of Motion (EOM) ansatz^85−87^ to describe electronically excited states through an excitation operator *R̂* (introduced below). This combination of the pCCD and EOM formalisms has been successfully employed in various studies of excited states in large molecular systems.^20,68,88−92^ The ansatz for the *k*-th excited-state wave function (within the pCCD approximation) reads
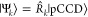
3Typically, the EOM ansatz should contain the same excitation operators as the cluster operator *T̂* in the chosen CC model (and the identity operator )
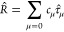
4where  labels some excitation operator from the occupied to the virtual orbital space (examples are given further below). However, since in pCCD the cluster operator is restricted to electron-pair excitations, the conventional EOM operator for electron excitations is restricted to the identity operator and all pair excitations^88,89^
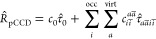
5In the above equation, we abbreviated the pair-excitation operator using . To obtain singly excited states, we use the EOM-pCCD+S model, which additionally includes single excitations in the *R̂* operator
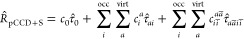
6Note that in the EOM-pCCD+S model, we target spin-free excited states where the single excitations are included using the singlet excitation operator . The electronically excited states are obtained by diagonalizing the similarity-transformed Hamiltonian of pCCD in the configurational space defined by *R̂*.^88,89,93^ Despite its simplicity, the EOM-pCCD+S excited state extension can reproduce excited-state properties that agree well with those predicted by the more elaborate and expensive linear response and EOM-CCSD variants.^82,92^ While the pCCD ground-state calculation features a computational cost of ,^34^ the computational scaling of the EOM-pCCD+S model increases to  (*o* indicates occupied, *v* virtual orbitals, and *N* = *o* + *v*).^82^ Nonetheless, both pCCD models are computationally more attractive than the CCSD counterparts, which feature a computational complexity of .

### 2.2 Domain-Based Charge Transfer Character

Charge-transfer dynamics can be systematically investigated by decomposing excited-state electronic configurations in molecular systems with defined electronic domains. Localized molecular orbitals allow us to uniquely establish such molecular domains. Excited states can then be characterized by tracking electron excitations between specific orbitals using configuration interaction (CI) vectors (see eq 6). By decomposing the excited state contributions across molecular domains, we can precisely map the electron transfer pathways. This involves examining the initial and final orbitals involved in electronic transitions, which allows us to determine the specific localized domains from which electrons originate and to which they are excited. In this work, we elaborate on a simple and intuitive charge-transfer decomposition analysis. Due to the localized nature of the natural pCCD-optimized orbitals, such a decomposition scheme can be straightforwardly and intuitively deduced from the excited-state wave functions.

**Figure 1:**
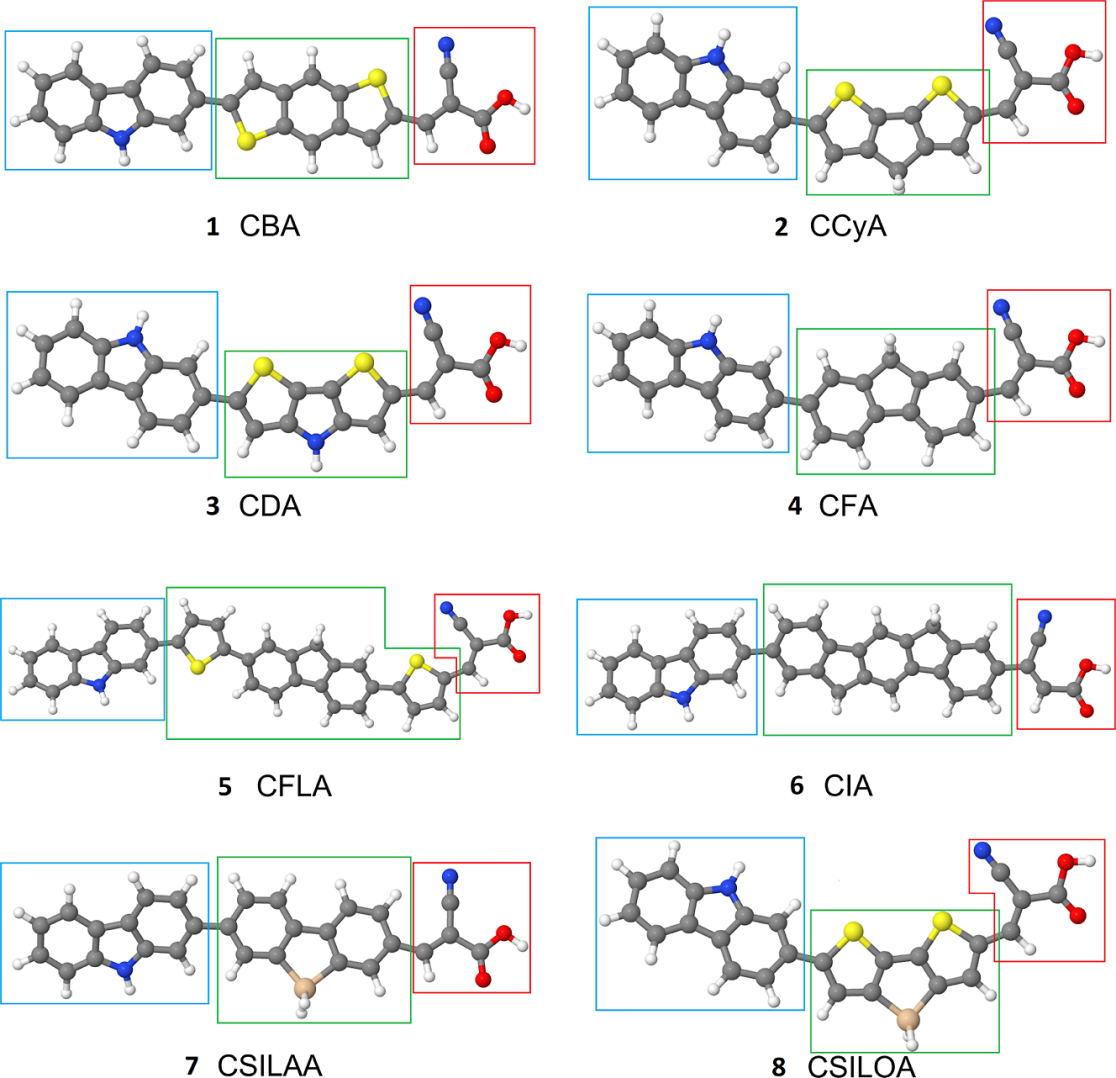
Molecular structures of the investigated dye molecules. The colors indicate different atoms: gray—carbon, white—hydrogen, blue—nitrogen, red—oxygen, yellow—sulfur, and beige—silicon. The colored boxes in the figure indicate different moieties: blue—donor, green—bridge, red—acceptor. 1: benzodithiophene bridge, 2: cyclopentadithiophene bridge, 3: dithienopyrrole bridge, 4: fluorene bridge, 5: fluorenebisthiophene bridge, 6: indenofluorene bridge, 7: silafluorene bridge, and 8: silolodithiophene bridge.

**Figure 2:**
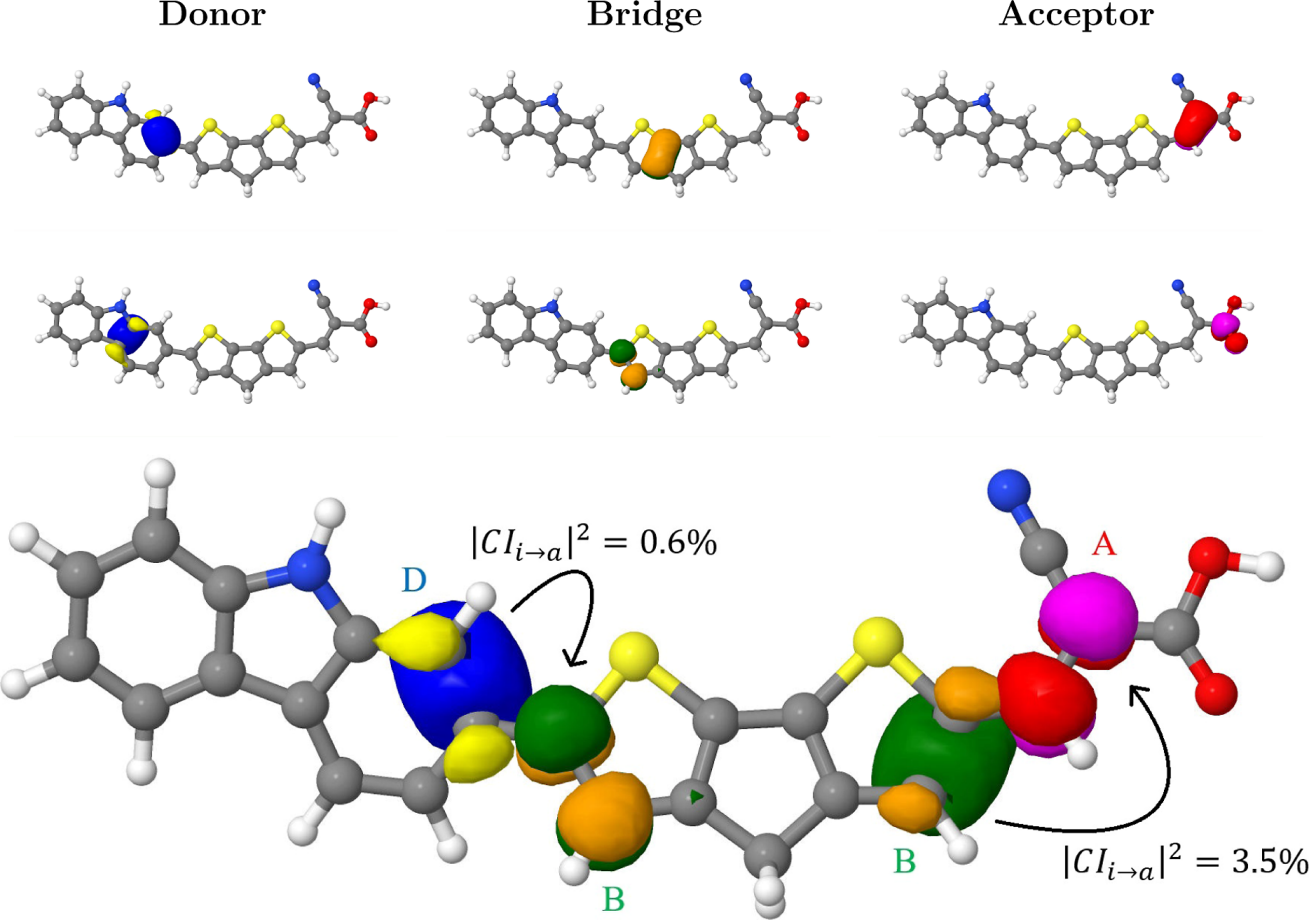
Graphical representation of the proposed domain-based charge transfer analysis. Top: isosurface plots of selected natural pCCD-optimized orbitals divided into the three investigated domains. Bottom: two terms entering eq 7 in the charge transfer analysis. Different domains are marked with letters and color schemes: A (red and magenta)—acceptor, B (orange and green)—bridge, D (yellow and blue)—donor. *i* → *a* stands for the transfer of one electron from the reference determinant (with occupied orbital *i*) to a virtual orbital *a*.

**Figure 3:**
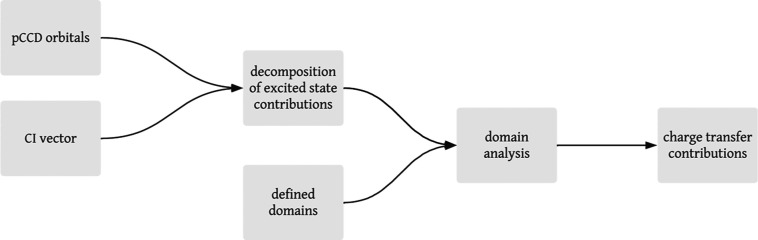
Flowchart summarizing the process of the charge transfer analysis. The first column marks the first step of the domain-based charge transfer decomposition (wave function optimization), the second column highlights the second step (domain assignment), while the right-hand side depicts the third step (charge transfer decomposition, evaluation of eq 7).

We emphasize that an in-depth analysis of electronic structure properties in extended organic electronic molecules is best understood in terms of localalized orbitals^94,95^ rather than canonical orbitals, which are delocalized over the entire system. The domains of each studied molecule can be seen in Figure 1 and an example of assigning orbitals to their corresponding domains is presented in Figure 2. The rest of the results, including an explicit assignment of orbitals to specific domains, can be found in the Supporting Information. Our strategy is summarized in Figure 3 and comprises three major steps. First, the ground- and excited-state wave functions need to be optimized. For that purpose, we perform an orbital-optimized pCCD ground state calculation^33,36,37^ to obtain a set of localized orbitals. The corresponding excited states are then optimized within some excited-state model, which yields the excited-state wave functions (the CI vectors in Figure 3). Second, we define molecular domains by grouping the localized natural pCCD-optimized orbitals into disjoint subsets. Specifically, each molecular orbital is associated with one domain/subspace. Since pCCD orbitals are commonly localized on at most two atomic centers, this domain-based assignment is straightforward (see Figure 2 for six such localized orbitals and their assignment to the donor, bridge, and acceptor domains). Molecular orbitals localized between two different domains are associated with one domain only. Here, we chose the domain based on the dominant atomic contributions from the Linear Combination of Molecular Orbitals in terms of Atomic Orbitals analysis. Third, the CI vector is decomposed into domain-based contributions. Specifically, each excited-state contribution is translated from the conventional occupied → virtual excitation to an initial domain → final domain character. The modulus squared of each domain-based CI vector element is taken and then summed over all domain-specific contributions. As an example, the bottom part of Figure 2 shows two excited state contributions, where electrons are excited between two different domains. Specifically, the left excitation contribution originates at the donor domain and goes to the bridge (CI_*i*→*a*_ translates to CI_donor→bridge_), while in the second one electrons are transferred from the bridge to the acceptor domains (CI_*i*→*a*_ translates to CI_bridge→acceptor_). Thus, the corresponding domain-based charge transfer character is determined from
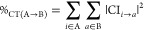
7where *A* and *B* label two disjoint domains (here, donor, bridge, and acceptor), and *i* and *a* label their corresponding molecular orbitals. CI_*i*→*a*_ marks one element of the excited-state vector, where *i* is some occupied orbital on domain A and *a* is some virtual orbital on domain B. These values are taken from the CI vectors of the EOM-pCCD+S excited state in question.

## 3 Computational Details

We employed various EOM approaches^85−87^ to deduce the orbital energies and electronic properties of a set containing eight dyes, displayed in Figure 1. Specifically, we used the Electron Affinity EOM (EA-EOM),^96,97^ Ionization Potential EOM (IP-EOM),^98−100^ and Electronic Excitation EOM (EE-EOM)^86,87^ variants combined with the pCCD and conventional CCSD approach.^101−104^ All IP-EOM-pCCD,^105^ EA-EOM-pCCD,^83^ and EE-EOM-pCCD with Singles correction (EOM-pCCD+S)^88,89^ calculations were performed with the Pythonic Black-box Electronic Structure Tool (PyBEST) v2.1.0.dev0 software package^106−108^ using GPU-acceleration.^109^ In all cases, the cc-pVDZ basis set^110^ was applied. The orbitals were optimized without any symmetry constraints using the variational orbital optimization protocol of pCCD as implemented in PyBEST.^33,37^ In all pCCD-based calculations, a frozen core approximation was applied. The number of frozen core orbitals applied in the pCCD calculations for each method was as follows: molecule **1** (36), molecule **2** (35), molecule **3** (35), molecule **4** (33), molecule **5** (47), molecule **6** (40), molecule **7** (35), and molecule **8** (37). The following threshold settings were used: pCCD energy threshold 10^–8^ (for orbital optimization), pCCD wave function threshold 10^–12^ (residual), orbital gradient threshold 5 × 10^–5^ (maximum absolute value), norm of orbital gradient threshold 10^–4^, EOM energy threshold 10^–8^, and EOM residual threshold 10^–5^. For a direct comparison, EEs, IPs, and EAs were calculated using the domain-based local pair natural orbital (DLPNO) scheme for the EE, IP, and EA variants of EOM-CCSD (EE/IP/EA-EOM-DLPNO–CCSD).^111,112^ These calculations were performed using the ORCA 5.0.4 software package using the “normal” PNO settings (*T*_cutPairs_ = 10^–4^, *T*_cutDO_ = 1 × 10^–2^, *T*_cutPNO_ = 3.33 × 10^–7^, *T*_cutMKN_ = 10^–3^, full iterative MP2 pair treatment).^113^ Calculations with “looser” and “tighter” threshold settings were also performed as sanity checks. Since we encountered memory issues for the “tighter” threshold, we will discuss numerical data from the “normal” PNO settings in the main text, while the remaining numerical results are collected in the Supporting Information. The domain-based charge transfer analysis (evaluation of eq 7) includes the lowest-lying excited state determined from EOM-pCCD+S calculations. When evaluating eq 7, we considered CI vector elements with |CI_(*i*→*j*)_|^2^ ≥ 10^–4^, while the overall %_CT(*A*→*B*)_ value included at least 93–95% of the excited state configurations (that is, elements of the CI vector).

All DFT calculations were preformed with the ADF2023^114,115^ program package using the TZ2P basis set.^115^ For the carbazole-based dyes, we investigated the following exchange–correlation functionals: PBE^116^ (GGA functional), PBE0^117^ (hybrid functional with 25% of HF exchange), CAM-B3LYP^118^ (range-separated hybrid exchange–correlation functional with 19% and 65% of HF exchange for the short and long-range, respectively), and the statistical average of orbital model potential (SAOP).^119^

Finally, we preformed test calculations to investigate solvation effects on excitation energies using DLPNO–CCSD and CAM-B3LYP within ORCA. In all these calculations a cc-pVDZ basis set was employed. We used vacuum structures and performed single-point calculations with two solvents: acrylonitrile and THF. The solvent was represented using the conductor-like polarizable continuum model (CPCM)^120^ as implemented in ORCA.

## 4 Numerical Results and Discussion

To illustrate the proposed charge transfer analysis, we investigate eight DSSC organic sensitizers, which share a common electron donor moiety based on a carbazole group and an electron acceptor component derived from cyanoacrylic acid. The eight dye molecules differ in their π-conjugated bridges, as depicted in Figure 1. We define their molecular domains as distinct moieties: a donor (blue boxes), a bridge (green boxes), and an acceptor (red boxes).

The investigated compounds incorporate a diverse range of π-bridges, including benzodithiophene (dye **1**), cyclopentadithiophene (dye **2**), dithienopyrrole (dye **3**), fluorene (dye **4**), fluorenebisthiophene (dye **5**), indenofluorene (dye **6**), silafluorene (dye **7**), and silolodithiophene (dye **8**). Although in dyes **4** and **6** bridges consist only of carbon and hydrogen atoms, molecules **1**, **2**, **3**, **5**, and **7** also incorporate sulfur atoms in their bridges. Dye **3** also features a nitrogen atom and dyes **7** and **8** are doped with one silicon atom. This structural diversity introduces variations in their electronic properties. These dyes have been the main focus of a previous study,^121^ which investigated the effect of various π-bridges on the optical and electronic properties of carbazole-based sensitizers for DSSCs using theoretical methods, including DFT. In their study, the authors analyzed the influence of different π-bridges on the absorption spectra, electronic structure, and charge transfer properties of the dyes. Thus, these molecules represent an ideal testing ground to check the validity and consistency of our domain-based charge-transfer decomposition and how the charge-transfer character evolves when changing the bridge in these dye candidates of DSSCs. Thus, our study on the domain-based charge transfer character and the IP/EA spectrum will provide additional insight into the electronic excitations and charge transfer characteristics that are crucial for light harvesting and charge separation in DSSC applications.

### 4.1 Comparison of Excitation Energies: pCCD vs CCSD and TF-DFT

Table 1 summarizes the lowest-lying excitation energies of all investigated molecules determined with the EOM-pCCD+S method, the EOM-DLPNO–CCSD variant, and selected methods within the time-dependent DFT (TD-DFT) framework. Most importantly, the excitation energies predicted by EOM-pCCD+S and EOM-DLPNO–CCSD exhibit remarkable agreement. This promising alignment bodes well for the validity of our approach. The DFT results generally follow trends similar to those observed in the pCCD analysis, with a few notable outliers, such as the exceptionally low result for molecule **8** calculated using CAM-B3LYP. The primary distinction lies in the fact that both the pCCD and DLPNO–CCSD methods yield consistently higher values than the DFT results across all molecules. Across all systems tested, the differences between the EOM-pCCD+S and EOM-DLPNO–CCSD methods remain small, typically less than 0.5 eV. For example, in the case of dye **2**, the excitation energies are nearly identical, with a difference of only 0.01 eV. Even for larger deviations, such as in dye **6** (0.50 eV) and **7** (0.38 eV), the overall trend remains consistent. This proximity of results indicates that the EOM-pCCD+S method, which leverages the pCCD reference function, effectively captures key electron correlation effects contributing to excitation energies.

**Table 1:** Comparison of the First Lowest-Lying Excitation Energies [eV] for Different Molecules Calculated Using the EOM-pCCD+S (*E*_pCCD_) and EOM-DLPNO–CCSD (*E*_DLPNO–CCSD_) Methods within the cc-pVDZ Basis Set and PBE, PBE0, CAM-B3LYP, and SAOP Methods within the TZ2P Basis Set^a^

molecule	*E*_pCCD_ [eV]	*E*_DLPNO–CCSD_ [eV]	|Δ_pCCD–DLPNO–CCSD_| [eV]	*E*_PBE_ [eV]	*E*_PBE0_ [eV]	*E*_CAM–B3LYP_ [eV]	*E*_SAOP_ [eV]
**1**	4.76	4.90	0.14	1.96	2.66	3.11	1.98
**2**	4.26	4.25	0.01	2.12	2.65	2.88	2.16
**3**	4.51	4.34	0.17	2.20	2.75	2.98	2.23
**4**	4.83	5.13	0.30	1.92	2.95	3.46	1.94
**5**	4.57	4.71	0.14	1.62	2.49	3.08	1.58
**6**	4.82	5.32	0.50	1.78	2.73	3.42	1.73
**7**	4.81	5.19	0.38	1.86	2.91	3.46	1.89
**8**	4.32	4.38	0.06	1.98	2.59	1.96	2.04

a Presented are also the differences between pCCD and DLPNO–CCSD results (|Δ_pCCD–DLPNO–CCSD_|).

Furthermore, the DLPNO–CCSD calculations were conducted with different threshold settings and varied solvents (see Supporting Information for numerical results and more details). Changing the PNO threshold settings results in a constant shift of about 0.4 to 0.5 eV in excitation energies. For tighter optimization settings, the excitation energies are lowered. We should note that one calculation with “tighter” PNO thresholds failed to converge due to memory limitations. Consequently, the “normal” PNO thresholds, for which all molecules converged, were chosen for this study. Accounting for solvation effects (more precisely, the CPCM^120^ solvation model) does not affect excitation energies (see Supporting Information for more details). Similarly, the CAM-B3LYP calculations were also performed with considering solvation effects. The inclusion of solvent does not affect the overall trends in excitation energies across the series of tested dyes but reduces the excitation energies by approximately 0.25 eV (see Supporting Information for more details). Hence, in the following, we will focus on comparing different methods in describing the electronic structures of the eight investigated dyes and the charge transfer character of the lowest-lying excited states.

The close agreement between the EOM-pCCD+S and DLPNO-EOM-CCSD excitation energies also suggests that the excited-state wave functions are well represented by the EOM-pCCD+S method. This observation is consistent with our previous findings,^82^ where the Linear Response formulation of EOM-pCCD+S and CCSD showed excellent agreement for excited state properties. Such a good agreement underlines the reliability and validity of the EOM-pCCD+S ansatz in describing electronic excitations, particularly in systems where strong electron correlation effects may dominate.

### 4.2 Charge Transfer Decomposition

**Figure 4:**
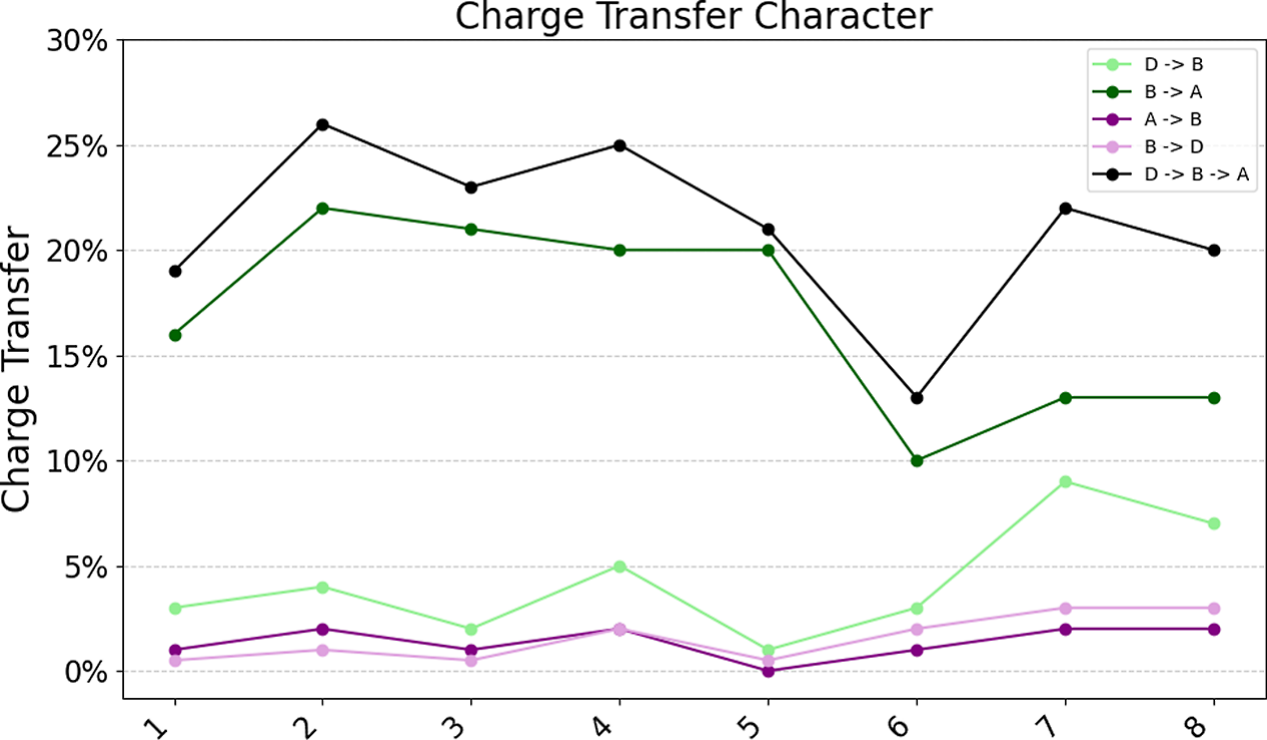
Percentage of the domain-based charge transfer character contributions %_CT_ (see eq 7) to the lowest-lying excited state of each dye **1**–**8** (EOM-pCCD+S/cc-pVDZ). Green lines indicate the desirable donor → bridge → acceptor (D → B and B → A) pathway, while purple lines mark the reversed direction acceptor → bridge → donor (A → B and B → D). The black line is the sum of the D → B and B → A %_CT_ values.

**Figure 5:**
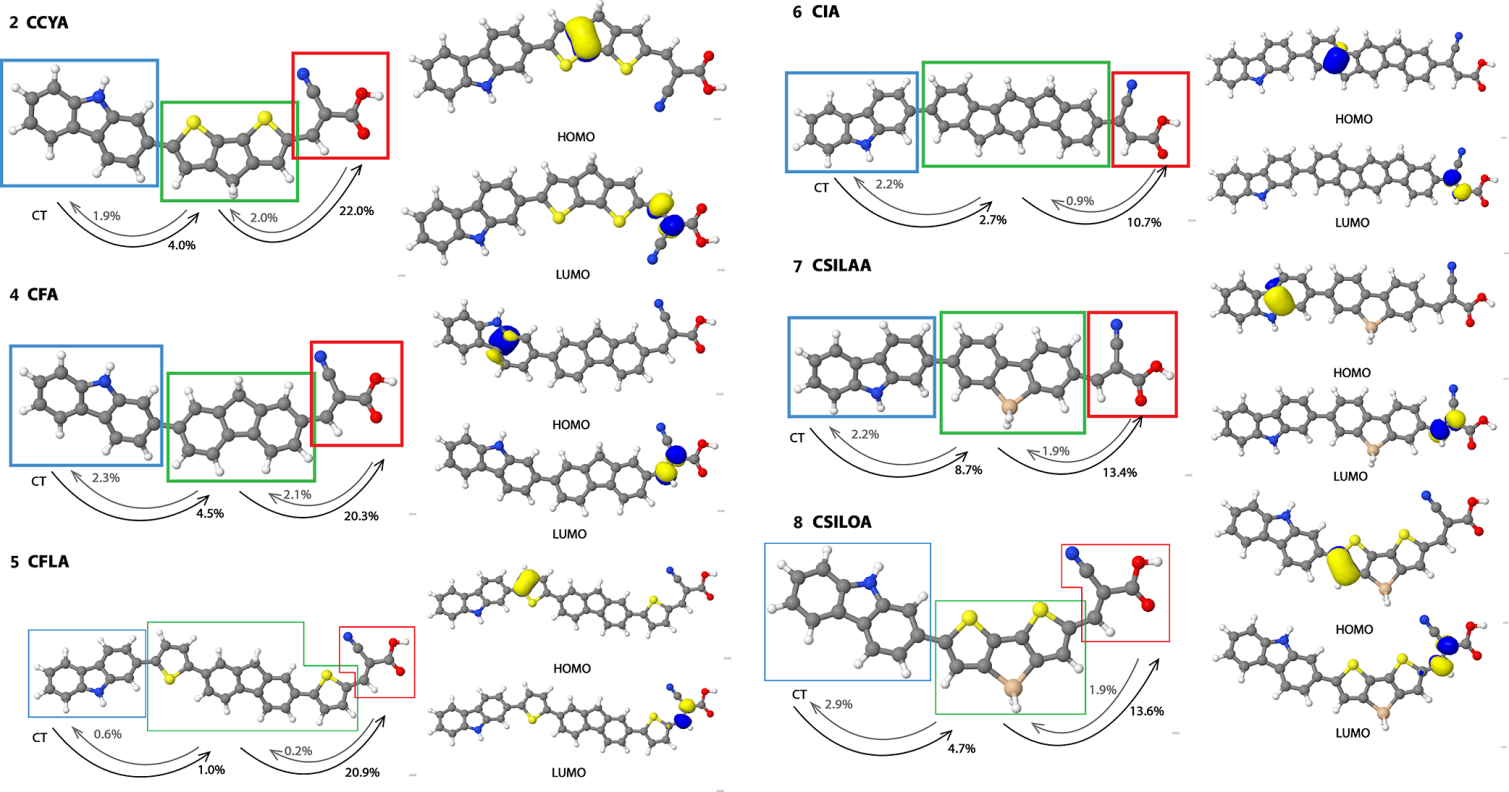
On the left side, the percentage of charge transfer character to the lowest-lying excited state for each dye. On the right side, pCCD HOMO and LUMO natural orbitals of the dyes. The colored boxes in the figure indicate different domains: blue—donor, green—bridge, red—acceptor. The pCCD HOMO and LUMO natural orbitals are identified as the dominant contributions to the IP and EA CI excited state vector.

Figure 5 and Table 2 display the domain-based charge-transfer character resolved with respect to the three defined domains (donor, bridge, and acceptor) for the investigated dyes. Specifically, we analyze the charge transfer in the direction donor → bridge → acceptor as well as the reverse pathway acceptor → bridge → donor. Figure 4 collects a pictorial representation of the domain-based charge-transfer decomposition, including the Highest Occupied Molecular Orbital (HOMO) and Lowest Unoccupied Molecular Orbital (LUMO), which in case of pCCD were obtained from IP-EOM and EA-EOM calculations, respectively. Our data on the charge-transfer contributions (see Table 2) reveal certain trends in how the structures of the molecules influence the efficiency of the desirable donor → bridge → acceptor pathway. High contributions to this pathway are essential for effective charge transfers in DSSCs. Our data also suggests that the bridge plays a critical role in either facilitating or hindering this transfer. The same charge-transfer (CT) analysis could not be performed for DLPNO–CCSD and DFT results as the orbitals obtained from these methods are not localized.

**Table 2:** Percentage of the Domain-Based Charge Transfer Character Contributions %_CT_ (See eq 7) to the Lowest-Lying Excited State of Each Dye **1**–**8** (EOM-pCCD+S/cc-pVDZ), Categorized by the Domain Orbitals (Initial Orbital → Final Orbital)^a^

	dye							
character	**1**	**2**	**3**	**4**	**5**	**6**	**7**	**8**
A → A*	0.061	0.075	0.072	0.092	0.010	0.025	0.075	0.045
A → B*	0.011	0.020	0.011	0.021	0.002	0.009	0.019	0.019
A → D*	0.000	0.000	0.000	0.000	0.000	0.000	0.000	0.008
B → A*	0.159	0.220	0.231	0.203	0.209	0.107	0.134	0.136
B → B*	0.416	0.537	0.403	0.475	0.685	0.685	0.494	0.633
B → D*	0.006	0.019	0.007	0.023	0.006	0.022	0.022	0.029
D → A*	0.000	0.007	0.000	0.007	0.000	0.001	0.040	0.004
D → B*	0.031	0.040	0.018	0.045	0.010	0.027	0.087	0.047
D → D*	0.032	0.036	0.006	0.075	0.014	0.056	0.070	0.033
**D → B → A**	**0.190**	**0.260**	**0.249**	**0.248**	**0.219**	**0.134**	**0.221**	**0.183**

a The orbitals are localized on the acceptor (A), donor (D), and bridge (B) domains. The * indicates the excitation to a virtual orbital of some domain with respect to the pCCD reference determinant. The last line D → B → A corresponds to the summed %_CT_ value of the D → B and B → A %_CT_ contributions.

Dye **2** (S-containing bridge) stands out for its most efficient donor → bridge → acceptor transfer, with contributions exceeding those of molecules **3** (S,N-containing bridge) and **4** (C-only bridge). The latter two, though slightly lower in their contributions, still exhibit high donor → bridge → acceptor charge transfer values. All three molecules have substantial bridge → acceptor contributions, highlighting the significant role of the bridge in charge transfer. Notably, dye **2** (S-containing bridge) and dye **3** (S,N-containing bridge) share a structural similarity, both incorporating the addition of sulfur atoms, which may influence their charge-transfer efficiency. Interestingly, other dyes containing sulfur, such as dye **1** (S-containing bridge), **5** (S-containing bridge), and **8** (S,Si-containing bridge), exhibit poorer performance, suggesting that sulfur alone does not guarantee better charge transfer properties.

Among the molecules examined, dye **6** (C-only bridge) emerges as the least efficient in terms of the donor → bridge → acceptor transfer. Its contributions are notably lower across all transitions, with weak donor → bridge and bridge → acceptor contributions. This suggests that the bridge plays a less effective role in facilitating charge transfer in molecule **6**, resulting in overall poor efficiency in this molecule. Interestingly, the undesirable charge transfer in this dye, as seen in the acceptor → bridge and bridge → donor pathways, does not exceed the levels observed in other molecules, as all molecules show similar acceptor → bridge → donor contributions below 5%. This observation indicates that the issue lies more with the donor → bridge → acceptor pathway rather than competing transitions.

Substituting a C atom with a Si atom (**4** → **7**) decreases the overall donor → bridge → acceptor charge transfer character (from 25% to 22%). An even stronger decrease is observed for the substitution of the N atom by a Si atom (**3** → **8**), where %_CT(D→B→A)_ reduces from 25% to 18%. Furthermore, extending the fluoren bridge in **4** by two additional thiophene groups (**5**) or by an indene group (**6**) lessens %_CT(D→B→A)_ from 25% to 22% and 13%, respectively. This trend suggests that extending the π-bridge with C-only groups has a reverse effect on the charge transfer character in these dyes. Finally, introducing Si atoms in the bridge generally reduces the %_CT(D→B→A)_ value across our testing series, while S- and N-doped bridges typically increase the %_CT(D→B→A)_ values.

The dihedral angle between the donor and bridge moieties is a critical factor influencing intramolecular charge transfer.^122^ A smaller dihedral angle, corresponding to greater planarity, facilitates more effective π -conjugation along the molecule, thereby enhancing the charge transfer process. This is consistent with the observation that the bridge and acceptor moieties exhibit substantial planarity, contributing to a higher charge transfer efficiency from the bridge to the acceptor. In contrast, the less planar configuration between the donor and bridge results in a reduced charge transfer efficiency. The planarity analysis suggests compounds **1**, **2**, **3**, **5**, and **8** as having the most favorable structural configurations for donor → bridge charge transfer. This is partially in accordance with our results.

In summary, the excitation type that predominantly occurs in the investigated dyes are bridge → bridge transitions, typically constituting 50% or more of the entire excitation process. Conversely, the direct donor → acceptor and acceptor → donor transitions contribute minimally.

### 4.3 IPs and EAs

**Figure 6:**
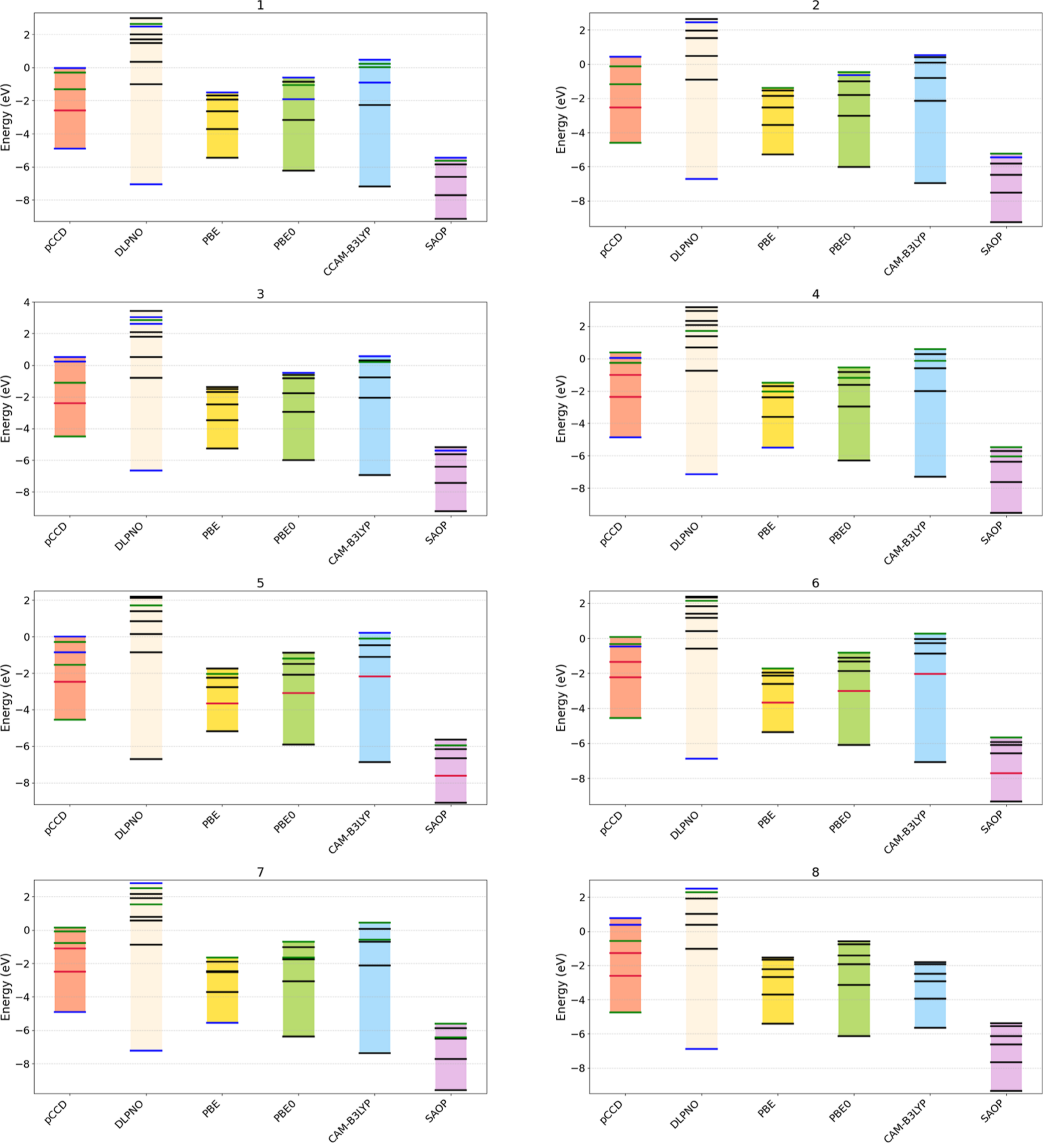
IPs and EAs of the investigated dye molecules calculated with pCCD, DLPNO–CCSD, PBE, PBE0, CAM-B3LYP, and SAOP. All IPs and EAs are displayed as—IPs and—EAs, respectively, to show the resemblance to the orbital energy spectra. The energy ranges are represented by colored floating bars, with specific energy levels indicated by horizontal lines. The lines correspond to the type of the moiety that shows the highest contribution (IP, EA, EA_1_, etc.). The color scheme represents different categories: black indicates delocalized orbitals, blue denotes donor orbitals, red signifies acceptor orbitals, and green marks bridge orbitals.

Figure 6 depicts the negative of IP and EA (or EA_*N*_) values determined from pCCD- and CCSD-type methods for each studied dye. For comparison, those values were also obtained with various approximate exchange–correlation functionals using Janak’s theorem.^123^ To clarify the key point, for the pCCD and DLPNO–CCSD methods, the IP and EA values were calculated directly, whereas for the DFT methods, they were approximated using the orbital energies. As in our charge transport analysis, we observe consistent trends with some notable exceptions in the IP and EA spectra between different computational methods. Across all methods, PBE systematically produces the narrowest energy spectrum, while DLPNO–CCSD yields the broadest spectrum, exhibiting a more pronounced separation. In particular, pCCD and CAM-B3LYP feature comparable EA levels, suggesting a shared capability to balance correlation and exchange effects. However, a certain difference emerges in the predicted IP values: pCCD systematically predicts slightly higher IPs compared to CAM-B3LYP. This observation is consistent with previous findings^66,124^ suggesting that the IP-EOM-pCCD method underestimates IP values by approximately 2 eV.

Furthermore, the delocalization and localization patterns of the molecular orbitals differ significantly between methods. For most cases, pCCD localizes the IP contributions on the bridge and the EA contributions on the acceptor (see Figure 5), whereas DFT methods (PBE, PBE0, CAM-B3LYP), as expected, tend to demonstrate delocalized orbitals. DLPNO–CCSD, while broadly delocalized, mostly localizes the IP contributions on the donor moiety.

We should stress that all methods yield quantitatively distinct energies, despite the similarities mentioned above. These discrepancies boil down to charge gaps differing up to a factor of 3 (DLPNO–CCSD vs PBE). On the other hand, all methods predict consistent changes in the energy spectrum when the bridge is modified. One exception is, CAM-B3LYP, where the energy spectrum of molecule **8** seems to be an outlier. In general, independent of the quantum chemistry method chosen, doping the bridge or extending its conjugation affects the relative IP/EA contributions’ positions and changes the resulting charge gap. Specifically, Si-doping of the bridge only marginally influences the charge gap (**2** → **8** or **4** → **7**), while N-doped compounds also show a downward shift of the energies (**2** → **3**). However, modifying/changing the bridge yields a more visible change in the charge gap (like **2** → **4**). Adding new doped groups decreases the charge gap (the negative value of IP shifts upward, the negative value of EAs downward, like **4** → **5**), while extending the C-only conjugation merely induces an upward shift as seen in the figure (like **4** → **6**). We should stress that these trends are predicted by all investigated methods. Notably, DLPNO–CCSD-type approaches result in larger band gaps than the corresponding pCCD-type variants, which has already been observed in previous studies.^83^ The charge gaps typically increase in the order PBE ≈ SAOP < PBE0 < pCCD < CAM-B3LYP < DLPNO–CCSD. The observed trends of energy levels and charge gaps when doping/extending the bridge are consistent with the changes in the domain-based charge-transfer character.

Finally, pCCD-type methods predict a similar electroaccepting power^125^ (a molecule’s ability to accept electrons) as DLPNO–CCSD-based methods, while large differences are observed for the electrodonating power^125^ (a molecule’s ability to donate electrons; see Figure S2 in the Supporting Information). In DSSCs, dyes should exhibit high electron-donating power to promote efficient charge injection and low electron-accepting power to minimize recombination and optimize device performance. In contrast to the DLPNO–CCSD data, the pCCD-predicted electrodonating power appears to be physically more sound. The resulting profiles of the electroaccepting and electrodonating powers predicted by pCCD variants suggest that dyes **2**, **3**, **5**, and **8** are promising dyes for DSSCs. Comparing these dyes’ electroaccepting and electrodonating powers to the donor → bridge → acceptor directed charge-transfer character suggests that molecules **2**, **3**, **4**, and—to a lesser extent—**8** feature satisfying properties as dyes in DSSC applications.

### 4.4 Comparison to Other Domain-Based Charge Transfer Analysis

While our methods provide valuable insights into charge transfer processes, different approaches can yield varying interpretations of electronic excitations. To ensure the reliability of our analysis, it is essential to compare our results with other established domain-based charge transfer analysis methods. This comparison allows us to assess the consistency of our findings, identify potential limitations, and highlight the strengths of our chosen methodology.

In order to do that, we performed a fragment decomposition analysis of the electron and hole populations of the excited states, as implemented in TheoDORE.^126^ This approach allows for a detailed characterization of the spatial distribution of charge within the molecule upon excitation, providing insight into the roles of different molecular fragments in charge transfer processes. We employed the CAM-B3LYP functional for these calculations, as it is well-suited for describing charge-transfer excitations. Furthermore, our choice of CAM-B3LYP is supported by its strong agreement with our computed IP/EA energies and excitation energies, reinforcing its suitability for this type of analysis. All numerical data regarding these comparisons can be found in the Supporting Information.

The molecular systems were divided into three fragments: donor, bridge, and acceptor, following the same partitioning scheme used in our previous pCCD-based studies. The results, summarized in Table S1 of the Supporting Information reveal distinct patterns in the distribution of electron and hole populations across these fragments. In all cases, the bridge fragment exhibits the highest hole and electron populations, indicating that the excitation is primarily localized in this region. The hole population on the bridge ranges from 0.635 to 0.834, while the electron population falls within the range of 0.395 to 0.693. This suggests that the bridge plays a dominant role in the excitation process, consistent with the findings from our pCCD calculations. The donor and acceptor fragments show smaller contributions, with hole populations varying between 0.070 and 0.240 in the donor and 0.065 to 0.164 in the acceptor. Similarly, the electron population remains lower in these regions, ranging from 0.037 to 0.133 for the donor and 0.259 to 0.568 for the acceptor. A graphical representation of these data further highlights the central role of the bridge fragment in the excitation process. As seen in Figure S4 of the Supporting Information, the charge redistribution upon excitation is largely confined to the bridge, reinforcing its function as the key domain facilitating charge transfer. These observations align well with our results obtained using pCCD-based methods, confirming the robustness of our approach.

In addition to the fragment decomposition of the electron and hole populations, an analysis was conducted to assess the relative hole and electron character of each molecular fragment. This was achieved by subtracting the hole population from the electron population. The resulting value provides insight into the dominant character of each fragment, with a positive value indicating a greater hole character, while a negative value suggests a greater electron character. The results of this analysis are presented in Figure S5 of the Supporting Information, which shows the hole–electron population differences for each molecular fragment in the excited states. The figure highlights that, as observed, the electrons are primarily located on the acceptor (negative values), while the holes are mostly distributed across the bridge and to a lesser extent on the donor (positive values). These findings are consistent with our data and the observed behavior of charge transfer in such systems, where the charge transfer is predominantly from the bridge to the acceptor.

A similar analysis was performed using our %_CT_ values, which exhibit good agreement with the results from the TheoDORE domain decomposition for molecules **2**, **3**, **5**, **7**, and **8**, as also shown in Figure S5 of the Supporting Information. Most of the molecules show consistent results across both methods, reinforcing the reliability of our analysis. For molecules **4** and **6**, however, discrepancies are observed. These molecules feature pure carbon bridges (without doping), and they also exhibit greater hole/electron character values compared to the doped counterparts. Such differences are likely due to the challenges associated with modeling π-conjugated systems within DFT, which are prone to delocalization errors. This is a well-known limitation of DFT. It is important to stress that despite the complexities associated with π-conjugated molecules and doped bridges, our method performs reliably, even for such challenging cases. Additional advantages of the pCCD-driven domain-based charge-transfer analysis are the absence of a population analysis or domain-specific optimization thresholds. Population analysis tools are typically strongly basis set dependent, while the tightness of the imposed optimization thresholds determines the accuracy of the localized domains. In pCCD calculations, the domains are defined through the orbital optimization, more specifically, by imposing the orbital gradient (eq 2) to vanish for both occupied and virtual orbitals. Setting the orbital gradient threshold to a large value is unsound as the corresponding orbital-optimized pCCD working equations are not fulfilled, and the corresponding lowest orbital-optimized pCCD wave function is not obtained. The actual influence of the basis set size on the orbital-optimized pCCD solution (both occupied and virtual orbitals) and the resulting charge-transfer character needs, however, to be assessed thoroughly, which is beyond the scope of the current work. These features highlight the robustness of our approach in accurately capturing charge transfer characteristics across a variety of molecular systems.

## 5 Conclusions

We have introduced a novel, intuitive, insightful, reliable, and computationally affordable approach for dissecting the charge transfer process in organic molecules. Our methodology is based on pCCD natural orbitals and their localized nature. This feature allows us to qualitatively and quantitatively monitor the charge transfer flow between different parts of organic molecules, such as donor and acceptor moieties, and the bridge between them. The analysis process is pretty simple and allows further use of more elaborate methods based on a pCCD reference function. Although time-consuming and repetitious, the analysis process can easily be fully automated. We demonstrated that the proposed domain-based charge-transfer decomposition can become a helpful tool to study potential DSSC components and other organic devices before diving into more expensive physical experiments.

Even though all dyes demonstrate high contributions to the desirable donor → bridge → acceptor charge transfer, our analysis points to molecules **2**, **3**, and **5** (and to a lesser extent **8**) as the finest candidates for DSSC applications. Our domain-based charge-transfer analysis and the pCCD-predicted electrodonating and electroaccepting powers confirm this observation. Moreover, dyes **2** and **3** have the advantage of having a relatively small charge gap. The former also retains a low excitation energy. This conclusion partially agrees with a previous DFT study^121^ that proposed molecules **5**, **8**, and **2** as the best candidates. Here, we extended the earlier DFT studies^121^ by examining different types of exchange–correlation functionals. Our work points to a strong predictive dependence of the orbital energies, and consequently the charge gap, on the chosen approximation of the exchange–correlation functional. Our results fall somewhere between the PBE0 and CAM-B3LYP ones, which should be considered the theoretically soundest (DFT) models due to their improved long-rage behavior.^23,127^ Our TheoDORE-based analysis also supports the findings of the pCCD and DFT-based methods, providing a detailed domain-based charge transfer analysis. It clearly identifies the bridge fragment as central to the excitation process, with the electron and hole populations being primarily localized on the donor and acceptor, respectively. This analysis corroborates the pCCD and DFT results, confirming that charge transfer is predominantly from the bridge to the acceptor. Notably, the results for molecules **2**, **3**, **5**, **7**, and **8** show strong consistency across methods, reinforcing their potential. We should stress that EOM-pCCD+S and the presented charge-transfer analysis provide consistent results for all investigated π-conjugated bridges (with and without doping). In contrast, CAM-B3LYP struggles with C-only π-conjugated bridges (due to delocalization errors) and the resulting change in hole and electron populations upon doping. Additional disadvantages of a DFT-based CT analysis are the strong basis set dependence of the underlying population analysis. Our pCCD-driven domain-based charge-transfer analysis does not require a population analysis to be performed. Instead, the localized domains emerge from the orbital-optimization condition of the orbital gradient, which must vanish in the case of the orbital-optimized pCCD states. The resulting pCCD-optimized orbitals are strongly localized. However, the basis set dependence of the pCCD-based domains remains an open question, which we aim to address in a systematic follow-up study.

Finally, having a good agreement between pCCD-based methods and the more elaborate DLPNO–CCSD variants for IPs, EAs, and electron excitations, we are convinced that our theoretical models have reliable predictive power. Simultaneously, the computational cost of the domain-based charge transfer decomposition does not exceed  (*o* indicates occupied, *v* virtual orbitals),^24^ which makes the investigated pCCD-based approaches promising candidates to study larger building blocks of organic devices. If need be, the accuracy of pCCD-based approaches can be further improved by including broken-pair states on top of the pCCD reference function by moving to some frozen-pair-type approach.^39,43,128^ Overall, our main goal of this work was to introduce a straightforward technique for describing the charge transfer character with localized pCCD-optimized orbitals. While our method demonstrates promise, the primary gap lies in the availability of theoretical reference data to benchmark our approach. Nonetheless, the change (increase or decrease) in the charge transfer character with respect to doping of the bridge with N, S, and Si atoms agrees with generally accepted experimental strategies to modulate electron-transfer properties in organic dyes and related compounds.^129^ Thus, the proposed domain-based charge transfer analysis exploiting the inexpensive EOM-pCCD+S approach represents an instructive tool to study large-scale systems where conventional DFT struggles. In follow-up works, we aim to address a fully automated version of the proposed domain-based charge-transfer analysis as well as to investigate the dependence of the molecular environment (like solvent), the molecular structure, or the underlying atomic basis set on the deduced charge-transfer character.

## Data Availability

The data underlying this study are available in the published article and its Supporting Information. The released version of the PyBEST code is available on Zenodo at https://zenodo.org/records/10069179 and on PyPI at https://pypi.org/project/pybest/.

## Supporting Information Available

SI.pdf: IP and EA spectra sorted with respect to methods, electroaccepting/donating powers determined for pCCD-type and DLPNO–CCSD-based methods, dependence of the DLPNO-EOM-CCSD results on different threshold values, effects of environment on the electronic spectra from TD-DFT and DLPNO-EOM-CCSD, and comparison of fragment decomposition of the electron and hole populations of the excited states exploiting CAM-B3LYP and EOM-pCCD+S, SI.xlsx: spreadsheets containing the domain-based charge transfer analysis, excitation energies, orbital energies, electrodonating and electroaccepting power (ZIP)
