# Supplementary material for: Domain-Based Charge-Transfer Decomposition and Its Application to Explore the Charge-Transfer Character in Prototypical Dyes
Source: J Chem Theory Comput. 2025 Apr 29;21(9):4506–19. doi: 10.1021/acs.jctc.5c00186 (PMC12079794; doi:10.1021/acs.jctc.5c00186)
Supplement: Supplementary file 1 — ct5c00186_si_001.zip [file ct5c00186_si_001.zip › si/SI.pdf]

A Domain-Based Charge-Transfer Decomposition and its Application to Explore the Charge-Transfer Character in Prototypical Dyes

Lena Szczuczko,[a] Marta Gałyńska,[b] Maximilian H. Kriebel,[a] Paweł Tecmer,[a*] and Katharina Boguslawski[a*]

[a] *Institute of Physics, Faculty of Physics, Astronomy and Informatics, Nicolaus Copernicus University in Toruń, Grudziądzka 5, 87-100 Toruń, Poland*

[b] *Faculty of Chemistry, Nicolaus Copernicus University in Toruń, Gagarina 7, 87-100 Toruń, Poland*

*Email: k.boguslawski@fizyka.umk.pl, ptecmer@fizyka.umk.pl

Supplementary Information

S1 IP and EA spectra

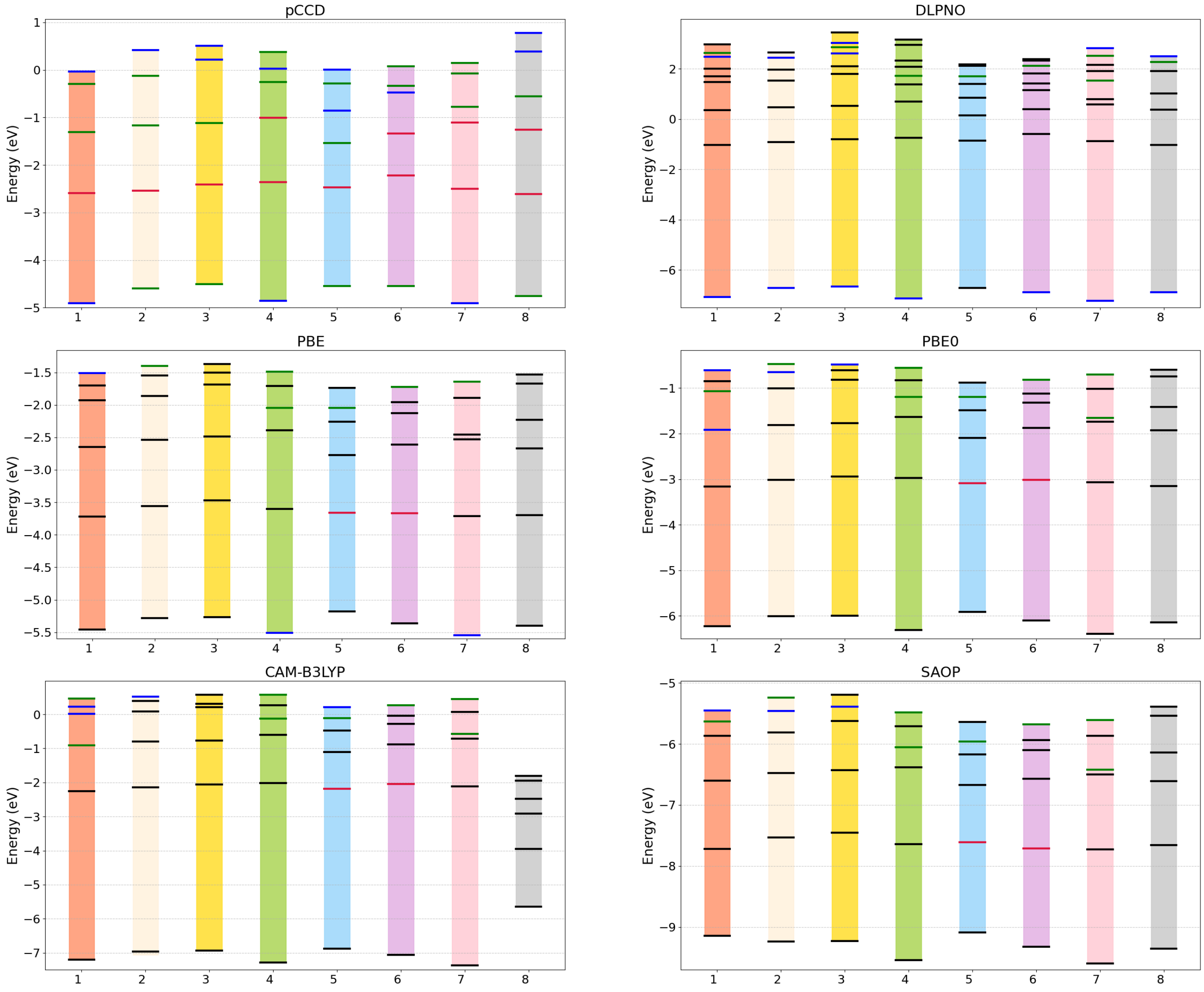

Figure S1: IPs and EAs of the investigated dye molecules calculated with pCCD, DLPNO-CCSD, PBE, PBE0, CAM-B3LYP, and SAOP. All IPs and EAs are displayed as −IPs and −EAs, respectively, to show the resemblance to the orbital energy spectra. The cc-pVDZ basis set was applied in all pCCD- and CCSD-based calculations. The TZ2P basis set was used for the DFT-based calculations. The energies are given in electronvolts (eV). Their ranges are represented by colored floating bars, with specific energy levels indicated by horizontal lines. The lines correspond to the moiety on which the given IP/EA is located. The color scheme represents different categories: black indicates delocalized orbitals, blue denotes donor orbitals, red signifies acceptor orbitals, and green marks bridge orbitals. This figure differs from Figure 6 in the main text, where the data is presented separately for each molecule to facilitate a comparison between methods. Here, we take the opposite approach, presenting separate graphs for each method to allow for a comparison among the molecules within a given computational approach.

S2 Electroaccepting and electrodonating powers

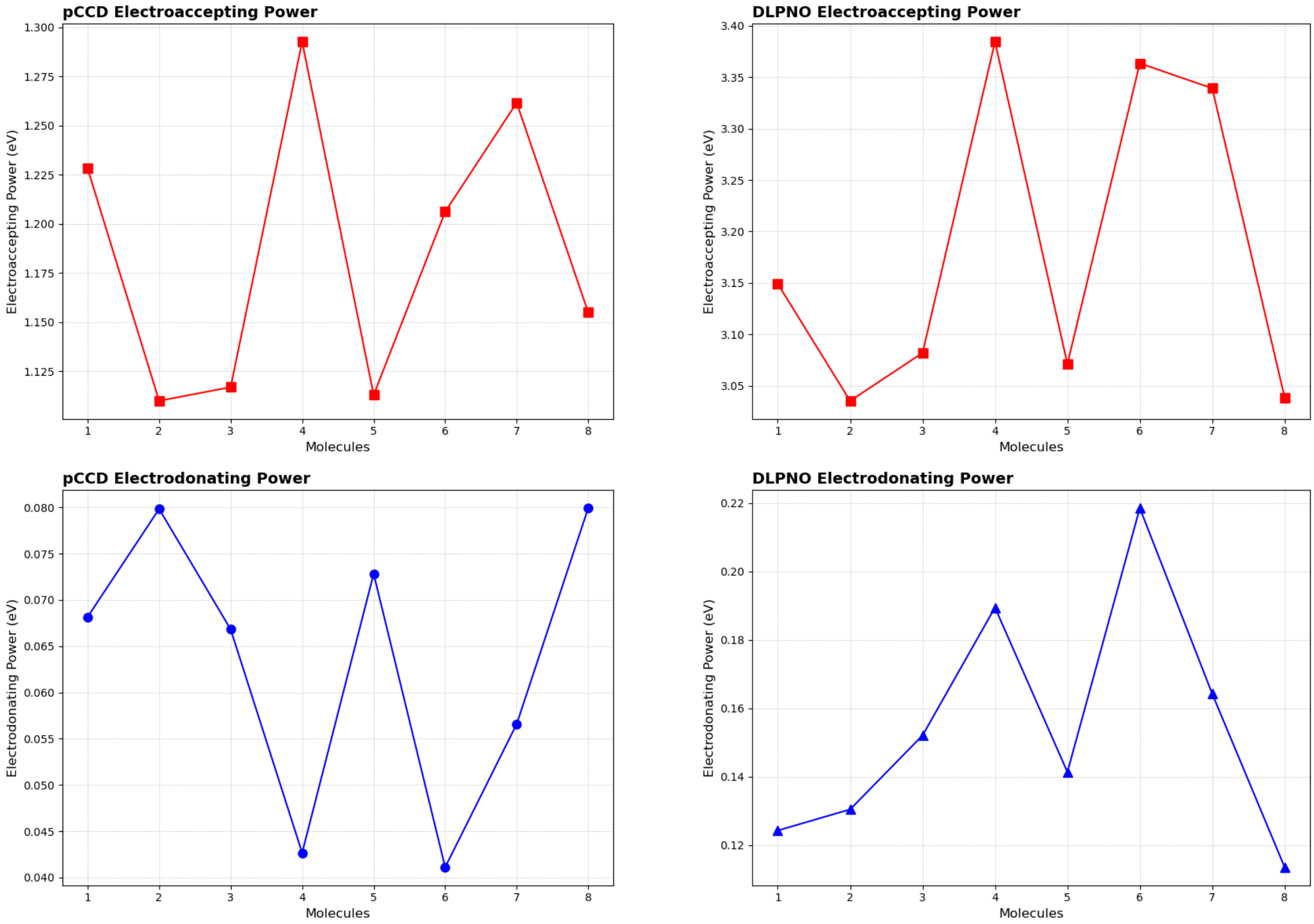

Figure S2: Electroaccepting (ω^+) and electrodonating (ω^-) powers of the molecular systems calculated with pCCD and DLPNO-CCSD methods, given in electronvolts (eV). All calculations used the cc-pVDZ basis set. These values are determined using the ionization potential (IP) and electron affinity (EA) according to the following equations: $\omega^+ = \frac{(IP+EA)^2}{8 \cdot IP}$, $\omega^- = \frac{(IP+EA)^2}{8 \cdot EA}$. Electroaccepting and electrodonating powers describe a molecule's ability to accept or donate electrons, respectively.

S3 Solvation effects and optimization thresholds

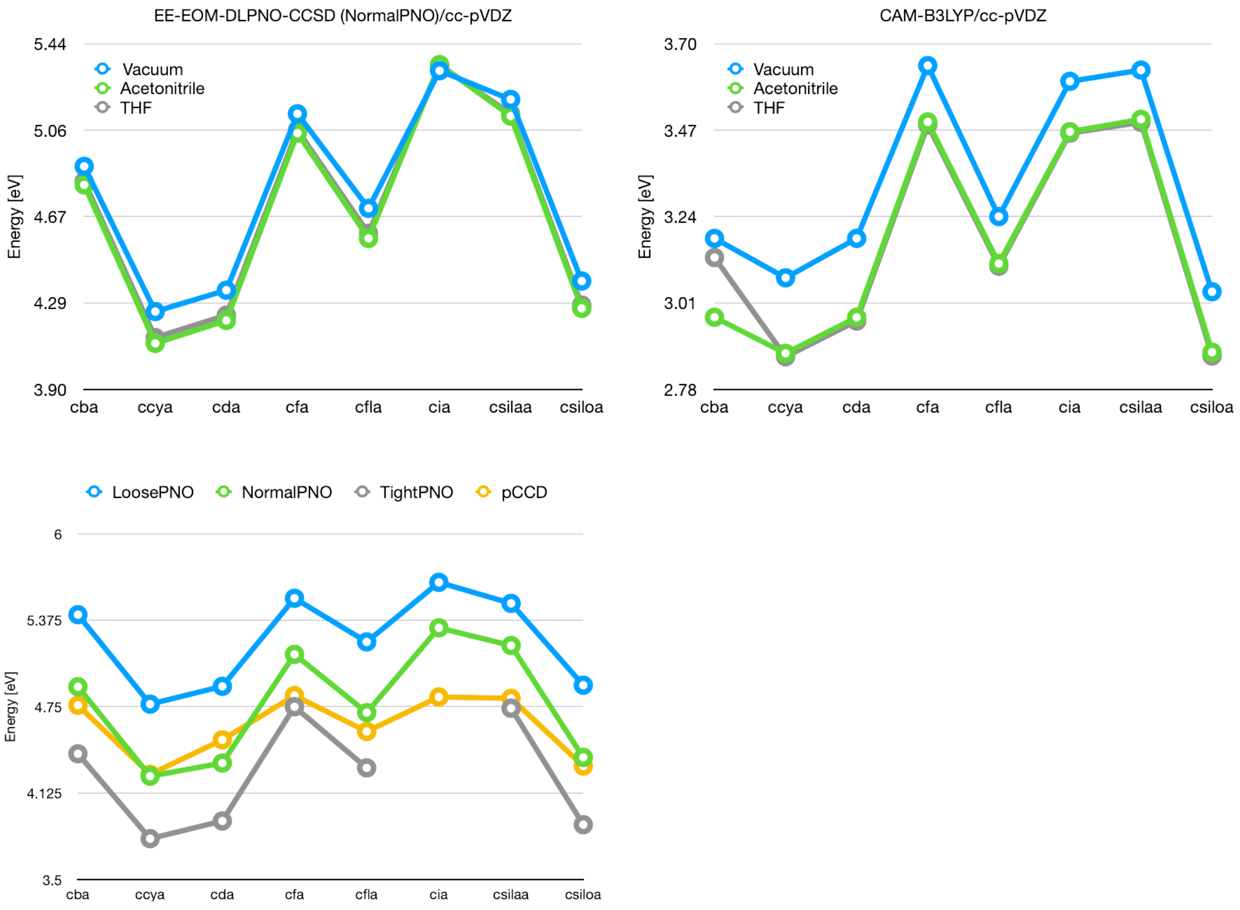

Figure S3: Lowest-lying singlet-singlet excitation energies calculated with different solvent using DLPNO-CCSD method (upper left) and CAM-B3LYP functional (upper right) and energies calculated with different DLPNO threshold settings (lower left). cba, ccya, cda, cfa, cfla, cia, csilaa, and csiloa corresponds to structures, **1**, **2**, **3**, **4**, **5**, **6**, **7**, and **8**, respectively. In all these calculations a cc-pVDZ basis set was used. We used vacuum structures and performed single-point calculations with two solvents: acrylonitrile and THF. The solvent was represented using the conductor-like polarizable continuum model (CPCM)[1] as implemented in ORCA. For one of the molecules, the calculations with tighter thresholds did not converge due to memory issues.

S4 Hole and electron decompositions

The presented domain-based decompositions obtained from the first excited state using EOM-pCCD+S were compared to results calculated with the CAM-B3LYP functional using a similar analysis: the fragment decomposition available in the TheoDORE software.[2] The fragments were defined in a similar manner, as shown in Figure 1 of the main text. The fragment decomposition analysis provides the populations of the hole and electron in specific fragments—in our case, the donor, bridge, and acceptor moieties. From this, we can easily deduce the dominant character of each moiety by subtracting the hole and electron populations belonging to each fragment. The hole and electron populations for CAM-B3LYP are summarized in Table S1 and Figure S4. The dominant hole and electron characters for CAM-B3LYP and EOM-pCCD+S are shown in Figure S5.

Table S1: The hole and electron decomposition analysis done for donor (D), bridge (B), and acceptor (A) for the first excited state of the investigated dye molecules calculated using CAM-B3LYP and EOM-pCCD+S.

	Hole_D	Electron_D	Hole_B	Electron_B	Hole_A	Electron_A
CAM-B3LYP						
1	0.165	0.092	0.753	0.529	0.082	0.379
2	0.200	0.121	0.645	0.573	0.155	0.306
3	0.194	0.133	0.643	0.512	0.164	0.355
4	0.199	0.037	0.656	0.395	0.145	0.568
5	0.070	0.042	0.826	0.693	0.104	0.265
6	0.101	0.052	0.834	0.530	0.065	0.418
7	0.240	0.088	0.635	0.566	0.125	0.346
8	0.207	0.099	0.657	0.642	0.136	0.259
EOM-pCCD+S						
1	0.063	0.038	0.581	0.458	0.072	0.220
2	0.083	0.055	0.776	0.597	0.095	0.302
3	0.024	0.013	0.641	0.432	0.083	0.303
4	0.127	0.098	0.701	0.541	0.113	0.302
5	0.024	0.020	0.900	0.697	0.012	0.219
6	0.084	0.078	0.814	0.721	0.034	0.133
7	0.197	0.092	0.650	0.600	0.094	0.249
8	0.084	0.070	0.798	0.699	0.072	0.185

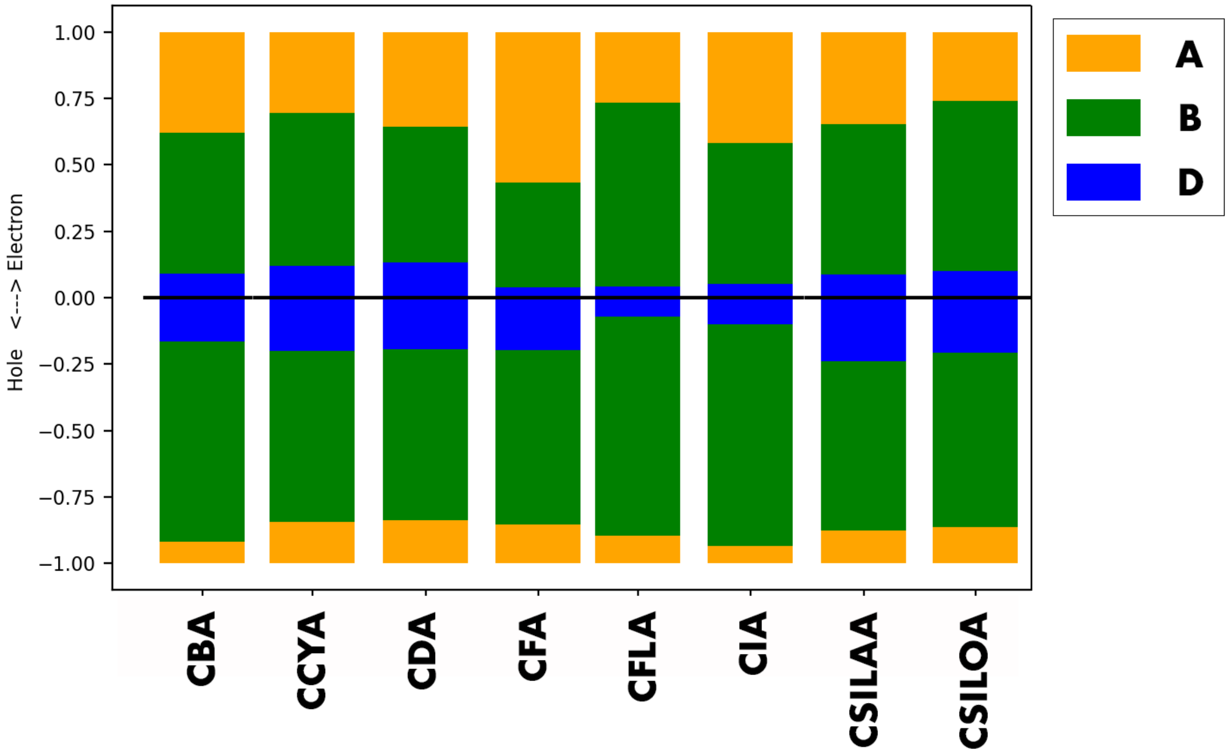

Figure S4: Contributions to the excitations based in the electron and hole population analysis using the CAM-B3LYP functional. The letters represent different molecular domains: acceptor (A), bridge (B), and donor (D). CBA, CCYA, CDA, CFA, CFLA, CIA, CSILAA, and CSILOA corresponds to structures, **1**, **2**, **3**, **4**, **5**, **6**, **7**, and **8**, respectively.

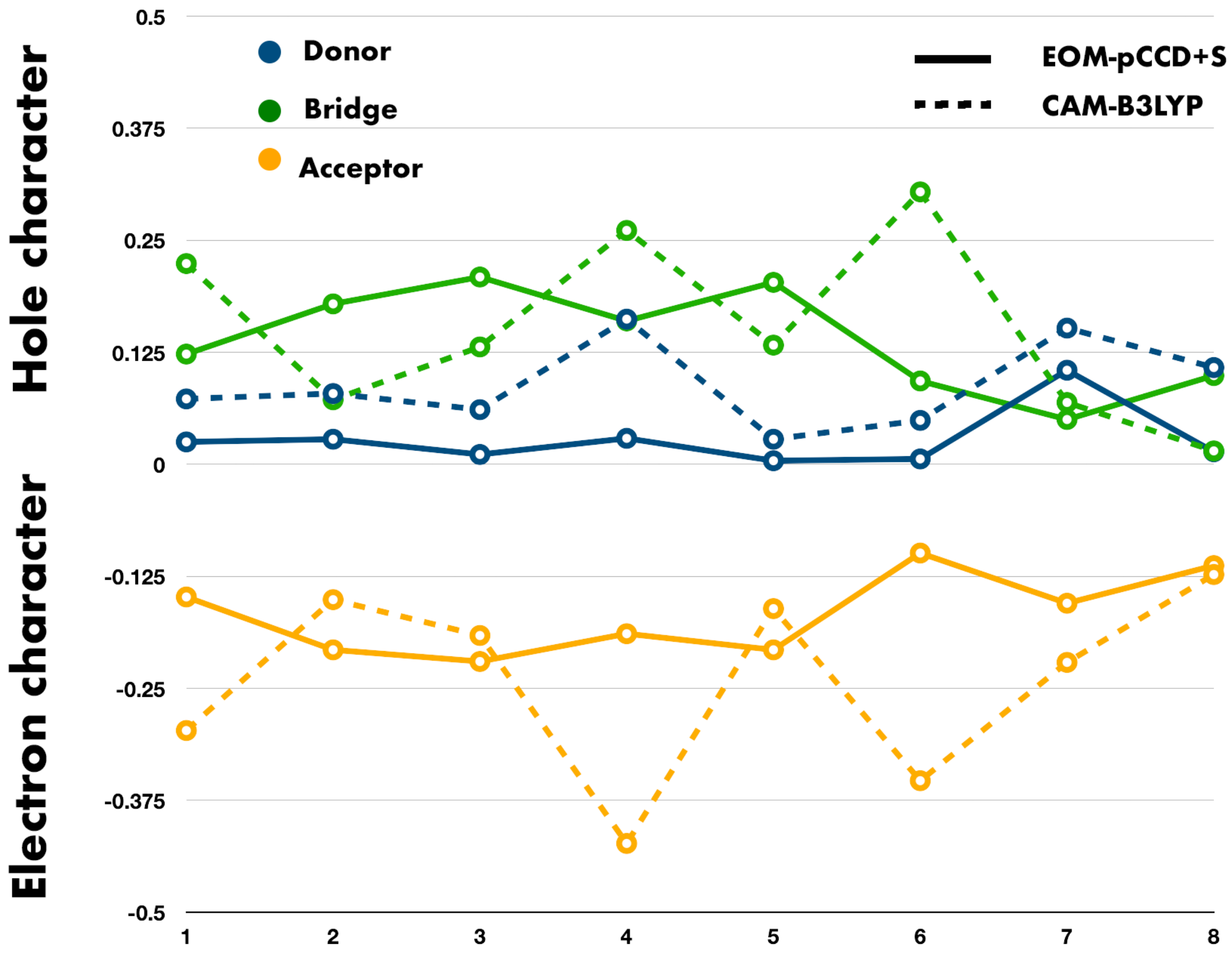

Figure S5: The hole and electron character for each domain (donor, bridge, and acceptor), obtained by subtracting the hole population from the electron population based on the CAM-B3LYP (dash line) and EOM-pCCD+S calculations. Positive values indicate a greater hole character, whereas negative values suggest a greater electron character.

References

[1] J. Tomasi, B. Mennucci and R. Cammi, *Chem. Rev.*, 2005, **105**, 2999–3094.

[2] F. Plasser, *J. Chem. Phys.*, 2020, **152**, 084108.
